# Long-term Outcomes After Open Repair of Humeral Avulsion of the Glenohumeral Ligament

**DOI:** 10.1177/23259671241247544

**Published:** 2024-06-01

**Authors:** Jasan Dannaway, Sumit Raniga, Desmond Bokor

**Affiliations:** † Faculty of Medicine, Health and Human Sciences, Macquarie University, New South Wales, Australia; Investigation performed at Faculty of Medicine, Health & Human Sciences, Macquarie University, New South Wales, Australia

**Keywords:** HAGL, shoulder instability, humeral avulsion of glenohumeral ligaments

## Abstract

**Background:**

There is a lack of data regarding the long-term clinical outcomes of open repair of humeral avulsion of the glenohumeral ligament (HAGL).

**Purpose:**

To examine the long-term patient outcomes, prevalence of related shoulder lesions, and return to sports in patients who have had open HAGL repair.

**Study Design:**

Case series; Level of evidence, 4.

**Methods:**

Included were 47 patients who underwent open repair of an HAGL lesion between 1995 and 2013. Clinical results were assessed using the Western Ontario Shoulder Instability Index (WOSI). Recurrence of instability, additional surgeries, confidence in the shoulder, level and type of sport before and after surgery, and return to sports were documented.

**Results:**

The mean follow-up duration was 105 months (range, 16-247 months). The mean postoperative WOSI score was 410. Postoperatively, 10 patients experienced a recurrence of instability. Subgroup analysis of patients who reported recurrence demonstrated significantly worse WOSI scores compared with patients who did not experience recurrence (730 [95% CI, 470-990] vs 320 [95% CI, 210-430], respectively; *P* = .007). Before surgery, 33 patients participated in competitive sports, compared with 22 patients after surgery. No postoperative neurologic or vascular complications were recorded. In 51% of patients, a labral tear was noted as a concomitant injury.

**Conclusion:**

Open repair of an HAGL lesion restored shoulder stability with good results. However, recurrence was significant (21%) with longer follow-up, and return to sports was affected. Associated lesions were prevalent.

Avulsion of the glenohumeral ligaments is associated with traumatic shoulder instability. Recurrent posttraumatic instability is often caused by lesions of the glenoid labrum, but less frequently, capsular or glenohumeral ligament injury can be the cause. In 1942, Nicola^13^ noted avulsion of the capsular ligaments in 4 of 5 patients who were operatively explored after acute dislocations. Little further research into capsular lesions was noted thereafter as focus turned toward Bankart lesions becoming established as the “essential lesion” responsible for glenohumeral instability.^17^ In 1988, Bach et al^1^ once again noted this pathology in their report of 2 cases of “lateral capsular avulsion” that were successfully managed surgically.

The term currently used for this lesion, “humeral avulsion of the glenohumeral ligament” (HAGL), was first used by Wolf et al^19^ in 1995. They presented a series of 64 patients with the diagnosis of anterior shoulder instability, whom they evaluated arthroscopically. Six (9.4%) shoulders were found to have HAGL lesions. They emphasized the importance of recognizing this lesion in the absence of a concurrent Bankart lesion and concluded that repair can restore anterior stability. In 1999, Bokor et al^3^ reported on a series of 547 shoulders undergoing operations for instability. In 41 (7.5%) shoulders, the cause of instability was deemed to be an HAGL lesion. Of the primary cases, the cause of the first dislocation was a violent injury in 33 (94.3%) shoulders, and 4 (11.4%) shoulders had a Bankart lesion. In patients whose first dislocation was a violent injury and who did not have a Bankart lesion and had no suggestion of multidirectional laxity, the incidence of HAGL was 39%. In 2019, Patzkowski et al^14^ reported HAGL lesions in 25% (9/36) of a cohort of female college athletes. An HAGL lesion is an established cause of shoulder instability, and a high index of suspicion is required, especially in cases in which associated lesions such as Bankart lesions are not present.

Likely because of the limited prevalence and detection of HAGL lesions, outcomes are often discussed in case reports or series. The majority of published HAGL repair series consist of <10 patients with a short duration of follow-up. Although the authors report a high rate of success in stabilizing the joint, they do not describe in depth the amount of return to activities, particularly in collision settings.

In 2016, Longo et al^11^ performed a systematic review and included 42 HAGL lesions, of which 25 were managed operatively. In 2017, Bozzo et al^4^ used a different search strategy and included 18 HAGL repair studies with 118 patients. Only 2 of the 79 patients managed surgically were unable to return to their prior levels of sport. The latest systematic review by Nelson et al^12^ in 2022 reported 117 surgically managed HAGL lesions, of which 60 were open procedures. Of these 60 cases, only 2 studies^6,15^ comprising 32 patients reported clinical outcomes. Only 1 of these studies,^6^ who included 15 patients, reported longer than midterm follow-up.

The literature suggests that arthroscopic or open surgical management of HAGL lesions provides comparable outcomes.^4^ However, open procedures enable visualization and may therefore improve the protection of nerves.^10^ Case reports have been published describing patients who received HAGL repairs and were unable to resume athletic activities given their limited range of motion. Castagna et al^5^ reported decreased internal rotation thought to be the result of excessive tightening in the posterior capsule. Schmiddem et al^18^ published a small case series in which 5 of 9 patients were unable to return to sports up to 59 months after surgery as a result of reduced external rotation, which might be attributed to the fact that 89% of patients had concurrent shoulder lesions.

The primary aim of this study was to provide long-term outcome data for open HAGL repair. The secondary aim was to provide an analysis of associated lesions and recurrent instability. This study is the first to assess long-term outcomes including return to sport and recurrence in a large cohort of patients who have had open HAGL repair.

## Methods

The study protocol received institutional review board approval. Patients were identified by retrospectively reviewing the practice database of the senior author (D.B.). All patients who had open HAGL repair between January 1995 and December 2013 were included. Before surgery, all patients underwent a clinical examination, standard radiographs of the shoulder, and dedicated imaging. All operations were performed by a single shoulder surgeon (D.B.). Surgical correction was performed on individuals with persistent instability after nonoperative treatment or those with a functional need for a stable shoulder. There were no conditions for exclusion and patients gave written consent.

Data were collated using a combination of the senior author's practice database and an electronic questionnaire. From the practice database, the patients’ characteristics, presenting symptoms, initial assessment (clinical examination, imaging modality, and findings), working diagnosis, intraoperative findings (including additional interventions), and surgical complications were collected. Patients were contacted by telephone and email in July 2017 to complete an electronic questionnaire. The questionnaire inquired about recurrence of instability, additional surgeries, confidence in the shoulder, level and type of sport before and after surgery, and return to sport. Furthermore, patients completed the Western Ontario Shoulder Instability Index (WOSI).

### Surgical Technique and Rehabilitation Protocol

All patients received an open repair of the HAGL lesion. The surgery was carried out according to a previously published technique.^3^ A deltopectoral approach was performed. Via a subscapularis tenotomy, careful blunt dissection of the capsule from the subscapularis was undertaken. Concomitant lesions in particular labral lesions were addressed as appropriate. The avulsed edge of the HAGL lesion was secured to the medial humeral neck using transosseous suture or suture anchors.

For 6 weeks, the operated shoulder was immobilized in a sling. Only active and passive motions of the elbow and wrist were permitted, as well as pendular shoulder movement, unless motion was restricted due to treatment of other injuries. In the sixth postoperative week, active range of motion exercises for the operated shoulder were started, and strengthening exercises began 3 months postoperatively. Six months postoperatively, a full return to unrestricted sporting activities was allowed.

### Statistical Analysis

The unpaired patient-reported outcome scores were nonnormally distributed. Therefore, comparisons of the patient-reported outcomes were conducted by Mann-Whitney *U* test. Means with 95% CI are graphically represented. Statistical analysis was carried out using SPSS (Version 23; IBM), and significance was set at *P* < .05.

## Results

This retrospective, single-surgeon cohort analysis comprised 47 patients who underwent open HAGL lesion repair between January 1995 and December 2013. The mean age of the patients at the time of injury was 24 years old (range, 13-58 years old), and the mean age of patients when contacted was 39 years old (range, 24-68 years old). There were 4 women and 43 men in the study sample. All patients had a history of a traumatic instability incident. The mean duration of follow-up was 105 months (range, 16-247 months). There were no recorded complications. Responses for questionnaires were sometimes incomplete. The number of responses for each section is detailed in the results to follow.

### Sports Participation

The group comprised a substantial number of professional collision athletes, none of whom had undergone shoulder surgery in the past. Before their injury, 19 patients participated in sports at a professional level (grade 1 or higher); 17 patients participated in rugby, 5 in Rugby League, and 24 in a range of other sports.

### Preoperative Examination

In total, 30 patients had undergone a magnetic resonance imaging study, and 4 received an magnetic resonance arthrogram. The information for the remaining patients was not recorded. The preoperative diagnosis of an HAGL lesion was only made in 22 (47%) patients. Of the remaining patients, 24 (51%) had a preoperative diagnosis of soft tissue Bankart injury and 1 (2%) had a bony Bankart injury. Where the preoperative imaging indicated there was an HAGL lesion, the repair was performed via an open procedure. Where preoperative imaging was inconclusive for an HAGL lesion, the diagnosis was made arthroscopically. After diagnosis, an open repair was performed.

### Intraoperative Findings/Associated Lesions

In 46 patients, there was an anterior HAGL lesion, and there was a combined anterior and posterior HAGL lesion in 1 patient. There was an anterior Bankart lesion in 23 individuals, a bony Bankart lesion in 2 patients, a rotator cuff tear in 3 patients, and a superior labral anterior-posterior (SLAP) lesion in 1 patient. In total, 23 Bankart repairs, 2 bony Bankart repairs, 1 SLAP repair, and 3 rotator cuff repairs were performed as supplementary procedures. The rotator cuff tears were traumatic in nature, and the mean age of these patients was 29 years.

### Functional Outcomes

Of the 47 patients, 41 responded to questions regarding confidence in their operative shoulder. A total of 29 patients reported recovering confidence in their shoulder after surgery. It took 16 patients 1 year, 9 patients 2 to 3 years, and 4 patients >3 years to achieve this. Twelve patients never acquired complete confidence in their shoulder. Postoperatively, 10 (21%) patients reported symptoms of instability at the final follow-up. Of these, 6 experienced dislocations, while 4 had subluxations. The mean duration between recurrence of instability was 55 months (range, 8-144 months). Only 2 of the 10 patients reported not regaining confidence in their shoulder before the subsequent instability event. All except 1 recurrent dislocation and 3 subluxations occurred with a new violent/collision event. Two patients had revision shoulder surgery (Table 1).

**Table 1:** Details of Recurrent Instability Events

Time From Surgery to Instability	Instability Type	Mechanism of Injury	Collision/Violent	Revision Surgery
8 mo	Dislocation	Throwing a ball	No	No
12 mo	Dislocation	Rugby union tackle	Yes	No
20 mo	Dislocation	Rugby union tackle	Yes	Yes
4 y	Subluxation	Martial arts throw	Yes	No
4 y	Subluxation	Throwing ball in soccer	No	No
5 y	Dislocation	Crash landing a hang glider	Yes	No
5 y	Subluxation	Reaching up fast to catch a ball	No	No
6 y	Dislocation	Diving to catch ball and landing in touch football	Yes	Yes
7 y	Subluxation	Reaching up fast to catch a ball	No	No
12 y	Dislocation	Diving to catch ball and landing in touch football	Yes	No

### Functional Scores

**Figure 1.**
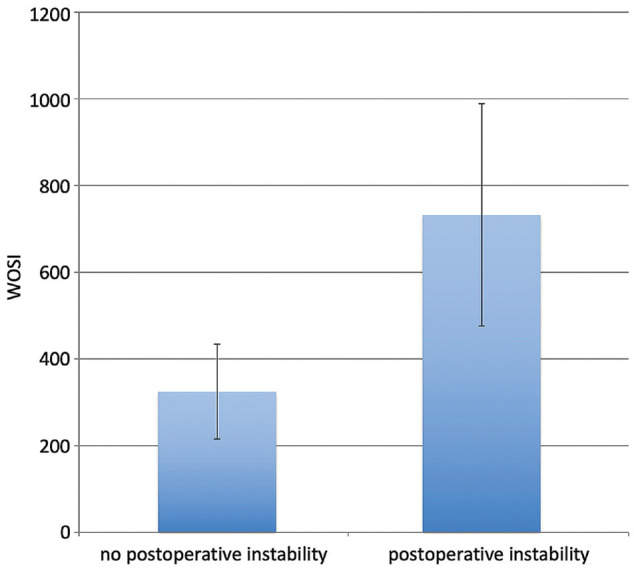
Mean Western Ontario Shoulder Instability Index (WOSI) scores of patients with and without postoperative instability. Error bars denote 95% CIs.

**Figure 2.**
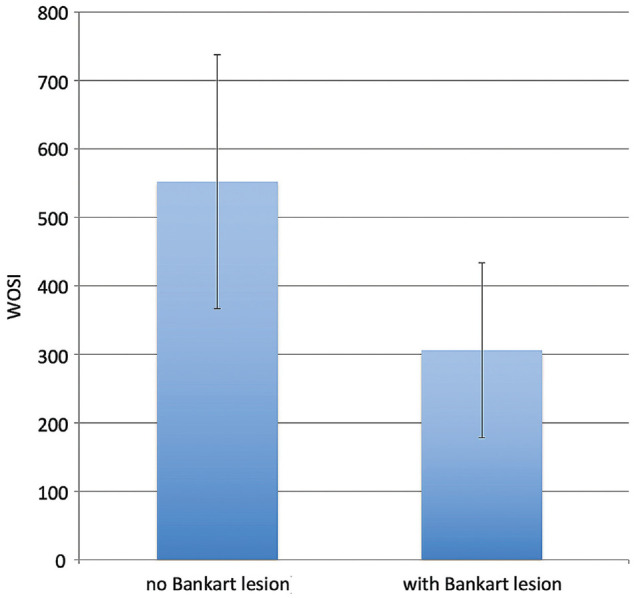
Mean Western Ontario Shoulder Instability Index (WOSI) scores of patients with and without Bankart lesions. Error bars denote 95% CIs.

Of the 47 patients, 41 completed the WOSI. The mean postoperative WOSI score was 410. WOSI scores were analyzed by subgroup according to patients with and without recurrence of instability, and those with and without Bankart lesions. Patients without postoperative recurrence had significantly better mean WOSI scores than those with recurrence (320 [95% CI, 210-430] vs 730 [95% CI, 470-990], respectively; *P* = .007) (Figure 1). As the minimal clinically important difference (MCID) of the WOSI is 294 points, this difference between the groups was also clinically significant. Individuals without a Bankart lesion had significantly worse mean WOSI scores than those with a Bankart lesion (550 [95% CI, 360-740] vs 310 [95% CI, 180-440], respectively; *P* = .02) (Figure 2). However, this may not be clinically significant, as the difference was less than the MCID.

### Return to Sports

Of the 47 patients, 39 reported participation in sports (competitive or social) before their shoulder injury; 33 patients participated in competitive sports, 19 of whom were professional athletes. Postoperatively, 40 of 47 patients reported participation in sports (competitive or social); 22 patients were involved in competitive sports, of whom 16 were competing professionally. Seven patients identified the surgical shoulder as the cause for their inability to return to sports. Two patients were unable to return to sports because of contralateral shoulder injuries, 2 to lower limb injuries, and 3 to unspecified injuries.

## Discussion

We reported the outcomes of a large series of patients, a large proportion of whom were collision athletes playing at a professional level, who underwent open HAGL repair. The reported outcomes were good; however, return to sports at the same level and confidence in the operated shoulder were affected. Postoperative dislocation mostly occurred with violent/collision environments and sometimes long after surgery. Recurrence of instability was not always the reason cited for stopping sport. This may indicate that open HAGL repair delivers a stable joint for return to sports; however, the resilience of the repair may be challenged by athletic exposures, just as other anatomical repairs are. There was a high incidence of associated lesions, mostly labral pathology. Preoperative diagnosis of HAGL was reasonably low. These findings indicate that a high index of suspicion preoperatively is required, particularly for patients with traumatic instability involved in high-risk collision sport and a magnetic resonance imaging study that does not show a Bankart lesion. If there is uncertainty, diagnostic arthroscopy may be useful.

Overall, the mean WOSI score at the final follow-up was 80% (410 points). WOSI scores were better when recurrence did not occur. Individuals without recurrence had a mean score of 85% (320 points), whereas those with recurrence had a score of 65% (730 points) (*P* = .007). This result is consistent with the findings of other authors, who have reported WOSI scores after HAGL repair of 88%^16^ and 91%^7^ when there was no recurrence. The WOSI score of individuals without a Bankart lesion compared with those with a Bankart lesion (550 vs 310, respectively; *P* = .02) was statistically but not clinically significant. This is supported by the literature,^6^ and it may suggest that clinical outcomes are dependent on identifying and addressing all lesions contributing to shoulder instability in order to achieve a stable shoulder. Furthermore, the presence of associated labral lesions does not confer poorer outcomes if addressed appropriately.

Recurrence of instability (subluxation and dislocation) occurred in 21% of patients. In their 2022 study, Davey et al^6^ reported a 20% (3/15) recurrence rate (subluxation and dislocation), with 7% (1/15) requiring revision surgery. Their cohort consisted of open HAGL repairs in competitive collision athletes, not dissimilar to ours, with a mean follow-up of 53.5 ± 17.4 months. Only 1 other case series^18^ that reported midterm follow-up (median, 59 months; range, 16-104 months) with recurrence looked at arthroscopically repaired HAGL lesions in a cohort of mostly competitive collision athletes. In this series, 7% (1/15) of patients experienced a recurrent dislocation. Recurrence in our series happened at a mean of 55 months (range, 8-144 months), and therefore, it is likely we have captured a greater number of these events. Furthermore, our series is larger and therefore likely more representative. Instability resulting from a new violent event is more a reflection of a high-risk environment than necessarily a failure of the surgical technique.

Twelve patients in our cohort never acquired complete confidence in their shoulder. Overall, participation in sports, social or competitive, remained similar pre- and postoperatively. Competitive sports participation declined from pre- to postoperatively (from 33/39 [85%] to 22/40 [55%]). Professional sports participation remained similar (preoperative, 19/39 [49%]; postoperative, 16/40 [40%]). Perhaps at the professional level, rehabilitation and other incentives to return to play explain the high return to sports in this group compared with the nonprofessional athlete. Only 7 patients identified the surgical shoulder as the cause of their inability to return to sports. Clearly, other issues arise in the high-risk group that influence return to sport. Davey et al^6^ reported return to the same level of sports in 12 of 15 (80%) patients and return to a lower level of sports in 2 of 15 (13%) patients. These data are similar to our results. Schmiddem et al,^18^ who performed arthroscopic repair, reported return to sports in 9 of 9 (100%) patients and return to the same level in 5 of 9 (56%) patients. There were a lower number of professional athletes in their study, and therefore, this supports our findings in the nonprofessional competitive athlete group. The overall mid- to longterm return-to-sports rate after the Latarjet procedure has been reported to be between 80% and 90%.^8,9^ Our results were comparable to this.

In our cohort, only 47% of HAGL lesions were diagnosed preoperatively. This suggests that in similar cohorts a high index of suspicion is required. Bokor et al^2^ outlined steps in order to reduce the chance of missing this diagnosis; however, no validated method has been proven in the literature. Associated lesions were very common (Bankart lesion, 49%; rotator cuff tear, 6%), which has been previously noted in the literature.^3,6^ This emphasizes the likely severity of the injury in this cohort.

### Limitations

Our study has multiple limitations. Its retrospective nature means that missing information (information bias) and recall bias need to be considered when interpreting the results. It was a single-surgeon cohort, which means that the external validity of our findings may be limited. We lacked a control group and preoperative patient-reported outcome scores, which limited our analysis. We treated a large number of associated lesions, which meant that assessment of HAGL repair in isolation was difficult. However, this pathology is relatively uncommon. We are not aware of a case series of this size reported in the literature to date. Furthermore, longterm follow-up has not been previously reported.

## Conclusion

The open repair of an HAGL lesion is a reliable, clinically effective way to restore shoulder stability. However, when significant follow-up is performed, recurrence is significant, and return to sport is affected. Preoperatively, HAGL lesions are underrecognized, and related lesions are frequent. The research available for comparison is limited, but our findings were, to some extent, consistent with the available literature. A multicenter collaborative investigation is the most likely way to further ascertain the outcome of these lesions.
